# Urban-Rural Health Transitions in India: A Comprehensive Review of Non-communicable Disease Trends and Risk Landscapes

**DOI:** 10.7759/cureus.90030

**Published:** 2025-08-13

**Authors:** Krupal J Joshi, Om Prakash Bera, Krishna M Jasani, Divyesh Gohel, Ankit Sheth

**Affiliations:** 1 Community and Family Medicine, All India Institute of Medical Sciences, Rajkot, IND; 2 Health Systems Strengthening, Global Health Advocacy Incubator, Washington, D.C., USA; 3 Pediatrics, Shantaba Medical College, Amreli, IND; 4 Health Sciences, Indian Council of Medical Research - National Institute of Occupational Health (ICMR-NIOH), Ahmedabad, IND

**Keywords:** diabetes mellitus, epidemiological transition, hypertension, non-communicable diseases (ncds), urban and rural disparities

## Abstract

India is experiencing a dual burden of non-communicable disease (NCD) risk factors, with distinct patterns emerging across rural and urban geographies. While behavioral risks such as tobacco and alcohol use remain entrenched in rural settings, urban populations are disproportionately affected by metabolic risks such as hypertension, diabetes, and obesity. This review aims to synthesize national data to understand the geographic divergence of NCD risk factors and their implications for public health policy. A comprehensive literature review was conducted using data from the National Family Health Surveys (NFHS-4 and NFHS-5), the National Noncommunicable Disease Monitoring Survey (NNMS), and the Indian Council of Medical Research - India Diabetes (ICMR-INDIAB) study. Risk factors were categorized into behavioral (e.g., tobacco use and alcohol consumption), metabolic (e.g., hypertension, overweight, and raised blood sugar), and biochemical (e.g., dyslipidemia) domains. Comparative analyses were performed across urban and rural populations to assess temporal trends and disparities. The review showed that rural populations demonstrated a higher prevalence of tobacco use (42.7% in rural men vs. 28.8% in urban men) and alcohol consumption, while urban populations had higher rates of physical inactivity, overweight, hypertension, and raised blood sugar. Obesity among rural women is also rising, indicating a shifting nutrition transition. Biochemical risk factors such as hypercholesterolemia were more prevalent in urban areas but remain underdiagnosed across both geographies. India’s NCD burden is characterized by a clear urban-rural divide in risk factor prevalence, necessitating geographically differentiated policy responses. Public health strategies must integrate behavior change in rural areas and metabolic risk management in urban settings, while addressing gender and equity gaps. Strengthening primary healthcare and aligning intersectoral efforts are crucial to achieving long-term NCD control and health equity.

## Introduction and background

India stands at a critical juncture in its health transition, grappling with an escalating burden of non-communicable diseases (NCDs) while still addressing longstanding challenges of communicable diseases and undernutrition. NCDs, including cardiovascular diseases (CVD), diabetes, cancers, and chronic respiratory illnesses, account for a growing share of morbidity and mortality [1]. In 2021, NCDs were responsible for approximately 74% of all deaths worldwide, with India accounting for an estimated 68% of this global mortality burden. Alarmingly, a substantial proportion of these deaths, about 55%, are classified as premature, occurring between the ages of 30 and 69 years, as reported in 2021 [2]. Of particular concern is the marked rise in the disability-adjusted life years (DALYs) associated with diabetes, which increased by over 80% between 2000 and 2019 [3]. This shift reflects broader socio-economic transformations, rapid urbanization, demographic transitions, and changes in lifestyle and environment [4]. However, the national discourse on NCDs often overlooks a crucial dimension: the growing disparity in NCD risk profiles between rural and urban populations.

Urban populations, with greater exposure to sedentary lifestyles, processed foods, and psychosocial stressors, are increasingly affected by metabolic and biochemical risk factors such as obesity, hypertension, and diabetes [5,6]. In contrast, rural areas continue to bear a disproportionately high burden of behavioral risk factors, including tobacco and alcohol use, low dietary diversity, and limited access to preventive health services [7,8]. These divergent patterns are not merely statistical; they reflect structural inequities in healthcare access, health literacy, regulatory enforcement, and socio-cultural norms.

Recent national datasets, including the National Family Health Surveys (NFHS-4 and NFHS-5), the National Noncommunicable Disease Monitoring Survey (NNMS), and the Indian Council of Medical Research - India Diabetes (ICMR-INDIAB) study, offer compelling evidence of these geographical differentials. Emerging trends also indicate a convergence of risks across the urban-rural continuum, with increasing overweight and obesity in rural women, and continued tobacco use in urban youth [9-12]. This convergence underscores the need for a more nuanced and context-responsive public health strategy that can tackle the dual and evolving burden of NCDs in India.

This review critically examines the urban-rural divide in NCD risk factors by analyzing behavioral, metabolic, and biochemical determinants through the lens of nationally representative data. It further proposes a conceptual and policy framework to address this divergence, advocating for differentiated, equity-oriented interventions within the National Programme for Prevention and Control of Non-Communicable Diseases (NP-NCD). The ultimate goal is to inform policy actors and practitioners on how to build more responsive, geographically tailored, and sustainable health systems for NCD prevention and control.

## Review

This review adopted a structured, narrative approach to examine the differential patterns of NCD risk factors across rural and urban populations in India. The methodology involved three key stages: data source selection, risk factor classification, and comparative thematic analysis.

Data sources

The review synthesized secondary data from nationally representative and peer-reviewed sources, including NFHS-4, 2015-16 [12]; NFHS-5, 2019-21 [9] for district- and state-level data on behavioral and metabolic risk factors. NFHS-4 surveyed 601,509 households and 699,686 women aged 15-49 years, while NFHS-5 increased the sample size to 636,699 households and 724,115 women aged 15-49. NNMS, 2017-18, [10] was used for population-level risk factor trends and health-seeking behavior. It had a sample size of 12,000 households, selected from 600 primary sampling units (300 rural and 300 urban) across 28 states and 348 districts in India. Within each household, one adult aged 18-69 years was selected. The ICMR-INDIAB study [11] was used for biochemical indicators and regional disparities in diabetes and related risks. These datasets were publicly accessible and were selected for their robust sampling designs, wide geographic coverage, and reliability in capturing sex-disaggregated and urban-rural stratified data.

Risk factor classification

There are several risk factors involved in the development of NCDs. As per the World Health Organization (WHO) STEPwise approach to surveillance (STEPS) [13], the risk factors were classified into three categories: behavioral, metabolic, and biochemical. Tobacco use, alcohol consumption, unhealthy dietary patterns, and physical inactivity constitute the behavioral factors. Metabolic risk factors include overweight/obesity, hypertension, and hyperglycemia. Biochemical risk factors include hypercholesterolemia and hypertriglyceridemia (where data were available). Each risk factor was analyzed based on prevalence, trends over time, and disparities between urban and rural populations.

Analytical framework

Risk Rating Matrix and Thematic Comparative Analysis

To assess and compare temporal and urban-rural variations in key NCD risk factors, a risk rating matrix was developed using data from three nationally representative datasets: NFHS-4 (2015-16), NNMS (2017-18), and NFHS-5 (2019-21). Five major NCD risk indicators were included: current tobacco use, current alcohol use, overweight/obesity, raised blood pressure, and raised blood sugar. Data were extracted for urban and rural populations and disaggregated by sex. Prevalence percentages were translated into risk categories using a three-tier system: low risk (<15%), moderate risk (15-29.9%), and high risk (≥30%), and visually coded as green, yellow, and red, respectively. These matrices enabled visualization of spatial, temporal, and gender-wise differences in risk profiles.

Complementing this, a comparative thematic analysis was employed to (i) identify disparities in the distribution of NCD risk factors across urban and rural contexts, (ii) detect convergence patterns such as the rising prevalence of overweight and obesity among rural women and the sustained behavioral risks (e.g., tobacco and alcohol use) in urban youth, and (iii) interpret the sociocultural, economic, and structural determinants underlying these patterns. This mixed-methods approach provided both quantitative assessment and qualitative interpretation of India’s shifting NCD risk landscape. Findings were synthesized into a conceptual framework and comparative matrix to inform context-specific policy recommendations.

Findings and review

Behavioral Risk Factors for NCDs

**Figure 1:**
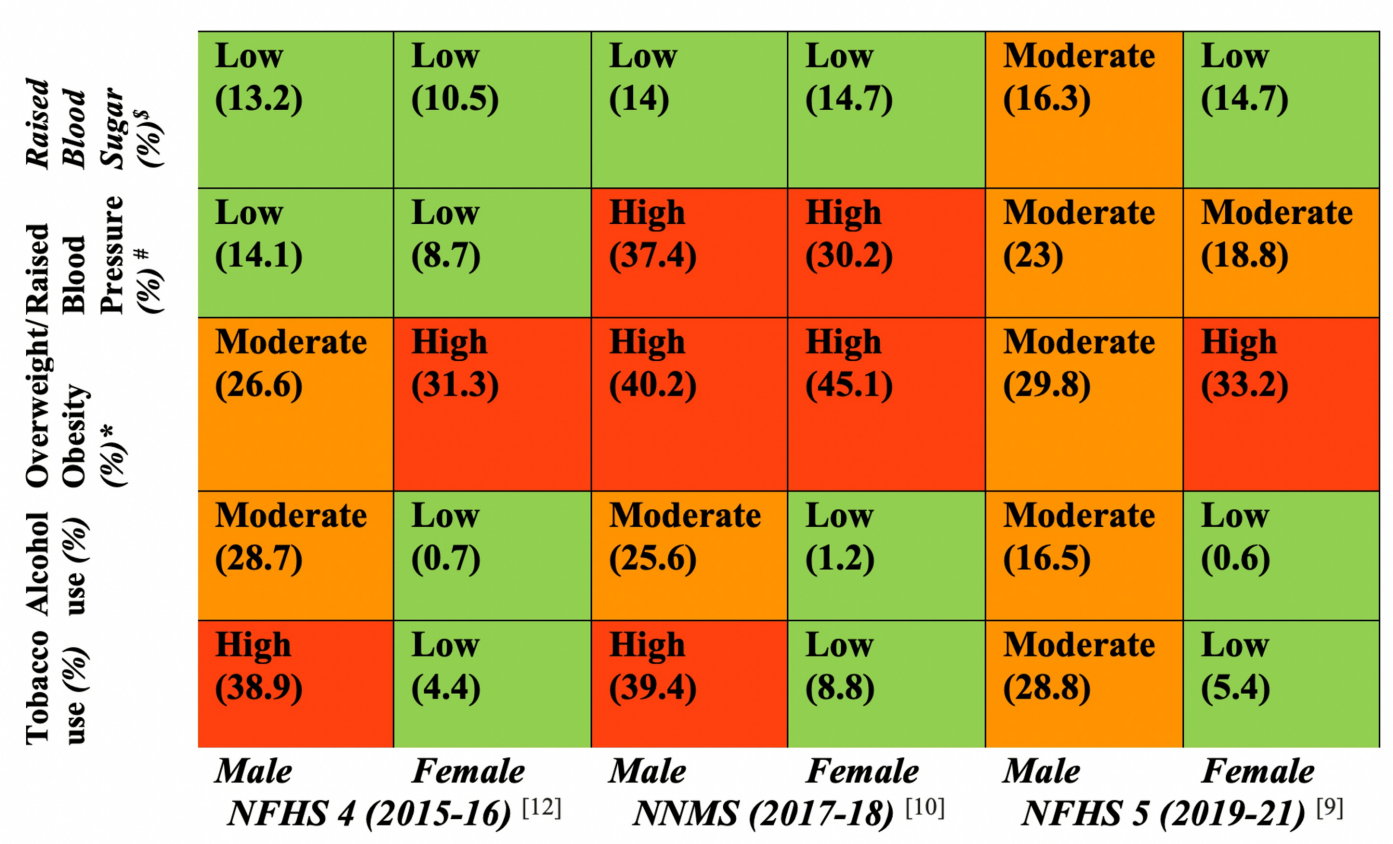
NCD risk rating matrix (urban) Figure 1 illustrates the urban NCD risk rating matrix derived from NFHS-4 (2015-16), NNMS (2017-18), and NFHS-5 (2019-21), comparing the prevalence of key NCD risk factors, raised blood sugar, raised blood pressure, overweight/obesity, alcohol use, and tobacco use, across time and gender. Each cell displays the risk category along with the corresponding prevalence percentage. The color codes represent the severity of risk: green indicates low risk, yellow/orange indicates moderate risk, and red indicates high risk. This matrix highlights temporal trends and gender disparities in urban NCD risk profiles, providing a visual summary to inform targeted public health interventions. *Overweight is defined as a BMI of ≥25 kg/m² in both the NFHS and NNMS surveys. ^#^Raised blood pressure is defined as a systolic blood pressure ≥140 mmHg and/or diastolic blood pressure ≥90 mmHg, consistent across both NFHS and NNMS surveys. ^$^Raised blood sugar is defined as a random blood glucose level ≥141 mg/dL in the NFHS surveys, whereas in the NNMS survey, it is defined as a fasting blood glucose level ≥126 mg/dL. NCD, non-communicable disease; NNMS, Noncommunicable Disease Monitoring Survey; NFHS, National Family Health Surveys Image Credits: Krishna M. Jasani

**Figure 2:**
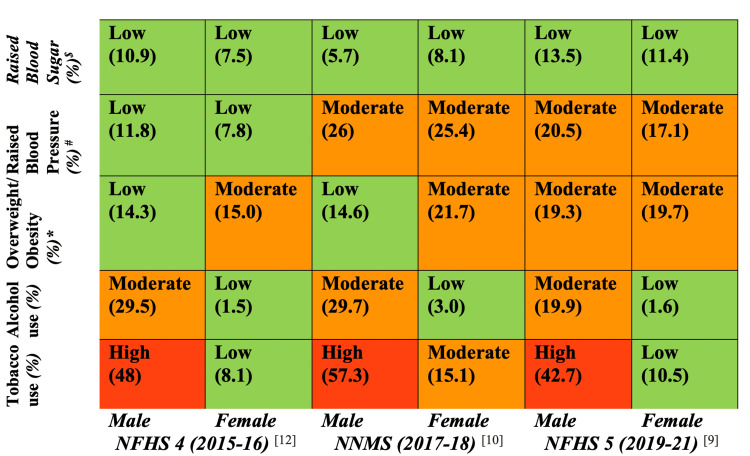
NCD risk rating matrix (rural) Figure 2 illustrates the rural NCD risk rating matrix derived from NFHS-4 (2015-16), NNMS (2017-18), and NFHS-5 (2019-21), comparing the prevalence of key NCD risk factors, raised blood sugar, raised blood pressure, overweight/obesity, alcohol use, and tobacco use, across time and gender. Each cell displays the risk category along with the corresponding prevalence percentage. The color codes represent the severity of risk: green indicates low risk, yellow/orange indicates moderate risk, and red indicates high risk. This matrix highlights temporal trends and gender disparities in rural NCD risk profiles, providing a visual summary to inform targeted public health interventions. *Overweight is defined as a BMI of ≥25 kg/m² in both the NFHS and NNMS surveys. ^#^Raised blood pressure is defined as a systolic blood pressure ≥140 mmHg and/or diastolic blood pressure ≥90 mmHg, consistent across both NFHS and NNMS surveys. ^$^Raised blood sugar is defined as a random blood glucose level ≥141 mg/dL in the NFHS surveys, whereas in the NNMS survey, it is defined as a fasting blood glucose level ≥126 mg/dL. NCD, non-communicable disease; NNMS, Noncommunicable Disease Monitoring Survey; NFHS, National Family Health Surveys Image Credits: Krishna M. Jasani

Tobacco use: Tobacco use remains a formidable public health challenge in India, contributing to an estimated 1.35 million deaths annually and serving as a major behavioral risk factor for NCDs. The dual burden of tobacco consumption in both smoked (e.g., bidis, cigarettes, and hookahs) and smokeless (e.g., khaini, gutkha, betel quid with tobacco, and zarda) forms complicates control efforts. Smokeless tobacco remains predominant, with nearly 267 million adult users, comprising approximately 29% of the adult population. Although tobacco smoking is comparatively less prevalent, it still affects over 10% of Indian adults. The total economic costs attributed to tobacco use from all diseases in India in 2017-18 for persons aged 35 years and above amounted to INR 177,341 crore (USD 27.5 billion) [14]. Disaggregated analyses across major national datasets, NFHS-4 (2015-16), NNMS (2017-18), and NFHS-5 (2019-21), reveal marked urban-rural differentials in tobacco consumption. In general, rural populations exhibit significantly higher tobacco use rates than their urban counterparts. According to NFHS-5, 42.7% of rural men and 10.5% of rural women reported current tobacco use, in contrast to 28.8% and 5.4% among urban men and women, respectively [9]. These trends are consistent with earlier findings from NNMS, which similarly highlighted rural predominance in both smoked and smokeless tobacco use (Figure 1 and Figure 2) [10]. Subnational data illustrate further variation in the magnitude of tobacco use across states. In Gujarat, NFHS-5 estimates indicate that 41.1% of men and 8.7% of women are current users of tobacco. Comparatively, GATS-2 (2016-17) reported prevalence figures of 35.5% among males and 10.4% among females in the state [15]. This persistence of high prevalence underscores the need for region-specific strategies, particularly in socioculturally diverse and economically stratified contexts. The urban-rural gap and regional disparities in tobacco use can be attributed to a complex interplay of demographic, socioeconomic, and cultural determinants. Evidence indicates that individuals with lower income, limited formal education, and those belonging to the Scheduled Castes and the Scheduled Tribes are more likely to engage in tobacco use [16,17]. Rural communities, in particular, often face barriers to cessation services, health literacy, and regulatory enforcement, which further perpetuate these disparities.

Harmful use of alcohol: The harmful use of alcohol remains a major global public health challenge, contributing an estimated 7.1% and 2.2% to the total burden of disease among males and females, respectively [18]. In the Indian context, alcohol consumption patterns and their associated health consequences exhibit marked disparities between urban and rural populations, influenced by complex cultural, socioeconomic, and regulatory factors. Harmful alcohol use is implicated in a range of adverse health outcomes, including liver cirrhosis, various malignancies, cardiovascular disorders, mental health conditions, and accidental injuries [19]. Nationally representative datasets, including NFHS-4, NNMS, and NFHS-5, consistently demonstrate a significantly higher prevalence of alcohol consumption among men compared to women, with a pronounced urban-rural divide. According to NFHS-5, current alcohol use was reported by 19.9% of rural men and 1.6% of rural women, compared to 16.5% and 0.6% among their urban counterparts, respectively (Figure 1 and Figure 2) [9,10,12]. While these figures represent a modest decline from previous estimates, rural areas continue to experience a disproportionately higher burden of harmful alcohol use.

This disparity is underpinned by a confluence of factors. In many rural and tribal communities, alcohol consumption enjoys sociocultural acceptance, often linked to traditional or ceremonial practices. Coupled with lower levels of health literacy, limited access to evidence-based interventions, and weak regulatory enforcement, these dynamics facilitate higher rates of consumption in rural settings. Moreover, the widespread availability of locally brewed, surrogate, or illicit alcoholic beverages, frequently contaminated with toxic substances such as methanol, further exacerbates health risks in these regions [18]. Importantly, the volume, frequency, and pattern of alcohol consumption are critical determinants of its health impacts. Higher lifetime consumption, recurrent binge drinking episodes, and early initiation are all associated with a significantly elevated risk of morbidity and mortality. Therefore, effective public health responses must adopt a context-sensitive approach that not only targets individual behavior change but also addresses the structural determinants of alcohol availability, affordability, and cultural normalization.

Physical inactivity: Physical inactivity is a well-recognized modifiable risk factor for NCDs, contributing to increased incidence of CVDs, diabetes, obesity, and certain cancers. Evidence from the NNMS (2017-18) highlights stark disparities in physical activity patterns across urban and rural India and between genders. Nationally, 41.3% of adults aged 18-69 years were classified as insufficiently active, with a notably higher prevalence among urban residents (51.7%) compared to those in rural areas (36.1%). Women consistently reported lower physical activity than men: 60.2% of urban women and 48.6% of rural women did not meet the recommended physical activity levels, compared to 44.2% of urban men and only 24.0% of rural men. Daily physical activity duration mirrored these patterns. On average, rural men engaged in 138.9 minutes of physical activity per day, more than three times that of urban women (41.7 minutes), who recorded the lowest mean activity. Rural populations overall had a higher average daily physical activity (101.1 minutes) than their urban counterparts (62.2 minutes). These discrepancies are influenced by differences in occupational demands, with rural individuals often engaged in labor-intensive agriculture, whereas urban lifestyles are increasingly sedentary. Correspondingly, sedentary time was highest among urban women (335.2 minutes/day), followed by urban men (314.8 minutes/day), further underscoring the risk posed by modern, inactive urban environments [10,20].

Unhealthy diet and nutrition: An unhealthy diet is a well-established modifiable risk factor for the development of major NCDs, including CVD, diabetes, and certain cancers. Globally, poor dietary quality ranks among the top contributors to overall disease burden. Evidence underscores the protective role of diets rich in vegetables, fruits, whole grains, legumes, and nuts, while limiting the intake of salt, free sugars, and unhealthy fats, particularly saturated and trans fats [21]. In India, the NNMS (2017-18) revealed that 98.4% of adults consumed insufficient amounts of fruits and vegetables, with slightly higher rates in rural (98.7%) compared to urban (97.7%) populations, and marginally more in women than men. In addition, high salt intake was observed, with mean daily consumption far exceeding WHO-recommended thresholds, averaging 8.0 g/day overall and as high as 9.2 g/day among urban men [10].

This nutritional crisis is further compounded by the increasing consumption of ultra-processed foods, driven by aggressive marketing, urbanization, and evolving dietary preferences [22]. Sharma et al. [21] highlighted that modern diets, especially in low- and middle-income countries, are increasingly dominated by energy-dense, nutrient-poor foods, escalating the risk of obesity and related NCDs. The issue is not unique to India; in high-income countries such as the United States, the problem has shifted from undernutrition to overconsumption, characterized by excessive intake of calories, saturated and trans fats, sugars, and sodium, accompanied by insufficient intake of key nutrients such as fiber, calcium, and vitamin D.

Metabolic and Biochemical Risk Factors

Overweight and obesity: The burden of overweight and obesity in India has been rising steadily over the past decades, with significant urban-rural and gender-specific variations. Data from three major national surveys, NFHS-4, NNMS, and NFHS-5, highlight the changing epidemiology of overweight in India. According to NFHS-4, the prevalence of overweight among urban males was 26.6%, compared to 14.3% in rural males. Among females, urban prevalence was even higher at 31.3%, versus 15% in rural areas [12]. By NFHS-5, urban overweight prevalence slightly decreased to 29.8% in males and 33.2% in females, while rural prevalence remained relatively stable at 19.3% in males and 19.7% in females [9]. The NNMS (2017-18) reported notably higher overweight rates in urban settings (40.2% in males and 45.1% in females) than in rural ones (14.6% and 21.7%, respectively), reaffirming the urban-rural divide (Figure 1 and Figure 2) [10].

This pattern reflects the lifestyle transitions associated with urbanization, such as decreased physical activity, increased consumption of calorie-dense foods, and greater sedentary behavior, which contribute significantly to the development of obesity-related NCDs [23,24]. Urban women, in particular, exhibit consistently higher prevalence rates, which may be attributed to cultural norms limiting physical activity, greater access to processed foods, and reproductive health factors such as postpartum weight retention [25]. However, the rural data also indicate a rising trend in overweight, particularly among women, suggesting that rural populations are undergoing a nutrition transition. The adoption of urban lifestyles, the mechanization of labor, and the increased availability of processed foods in rural markets are narrowing the traditional urban-rural health divide [26]. A multicentric study by Geldsetzer et al. [8] using national survey data reported that overweight and obesity prevalence are increasing more rapidly in rural than in urban populations, both in relative and absolute terms, especially in lower- and middle-income countries like India. Similarly, the ICMR-INDIAB study found a significant prevalence of obesity even in tier 2 and 3 cities and rural areas, calling attention to the pervasiveness of this risk factor across geographic settings [11]. Given the association of overweight and obesity with raised blood pressure and blood sugar, both of which also show higher prevalence in urban areas as per the NFHS and NNMS, it is evident that addressing overweight is central to NCD prevention and control. Integrated strategies, including behavior change communication, promotion of physical activity, taxation of sugar-sweetened beverages, and urban design fostering walkability and active transport, have been recommended by WHO and should be tailored to India's dual-burden context [27].

Raised blood pressure: An analysis of nationally representative surveys, NFHS-4, NFHS-5, and NNMS, reveals a concerning rise in the prevalence of raised blood pressure among Indian adults, accompanied by shifting urban-rural patterns. Between NFHS-4 and NFHS-5, prevalence increased significantly: from 14.1% to 23.0% in urban males and from 8.7% to 18.8% in urban females, while rural males and females saw a rise from 11.8% to 20.5% and 7.8% to 17.1%, respectively [9]. This narrowing urban-rural gap indicates a broader epidemiological transition, likely driven by the diffusion of sedentary lifestyles, poor dietary habits, and rising obesity in rural areas. The NNMS reported even higher prevalence, 37.4% among urban males, 30.2% among urban females, and 26.0% and 25.4% among rural males and females (Figure 1 and Figure 2) [10]. Notably, evidence suggests that urbanization contributes disproportionately to NCD risk among women compared to men [28], while rural-to-urban migration has been associated with increased obesity, elevated blood pressure, lipid abnormalities, and higher fasting glucose levels, particularly among men [29]. Studies further indicate that altered diets and reduced physical activity in urban middle-class males significantly elevate conventional CVD risk factors [30].

Additionally, physical inactivity, increasingly common in both urban and rural contexts, particularly due to mechanization and changing work environments, plays a central role in blood pressure elevation. Tobacco and alcohol use, prevalent in rural males as shown in NFHS data, also contribute significantly to hypertensive risk [9]. The role of early-life undernutrition and intrauterine growth restriction, leading to developmental origins of hypertension, is particularly relevant in rural India, where childhood malnutrition remains high [31]. Moreover, social determinants such as low health literacy, inadequate access to preventive care, poor treatment adherence, and socioeconomic stressors further compound the burden. Studies by Geldsetzer et al. [8] and Anchala et al. [32] have underscored these interlinked challenges, revealing major gaps in hypertension awareness, treatment, and control, particularly in rural and underserved areas.

Raised blood sugar: The data reveal a concerning upward trend in the prevalence of raised blood sugar levels across India between NFHS-4 and NFHS-5, with urban males showing an increase from 13.2% to 16.3%, urban females from 10.5% to 14.7%, rural males from 10.9% to 13.5%, and rural females from 7.5% to 11.4% [9,12]. NNMS data echoed this urban predominance, reporting raised fasting blood glucose (≥126 mg/dL) in 14.0% of urban males and 14.7% of urban females, whereas rural males and females reported lower prevalence (5.7% and 8.1%, respectively) (Figure 1 and Figure 2) [10]. Raised blood glucose levels have been documented for about 17.7% (223,986,000) of the Indian population by the ICMR INDIAB (ICMR, 2016) survey [11]. The International Diabetes Federation's (IDF) 2021 report shows the overall prevalence of diabetes among adults aged 20-79 in India at 8.3% (7.3 to 9.3%), slightly lower than the global prevalence of 10.5% [33]. This nationwide escalation likely stems from multiple interacting risk factors, including significant dietary changes characterized by increased consumption of processed foods and sugar-sweetened beverages in both urban and rural areas, coupled with declining physical activity levels due to urbanization, mechanization of agriculture, and more sedentary occupations. The parallel rise in obesity rates, particularly abdominal adiposity, further compounds diabetes risk, while demographic aging expands the at-risk population [34,35]. Notably, the sharper relative increase among rural females suggests they may be losing traditional protective factors like active agricultural lifestyles and whole-food diets. Additional contributors may include psychosocial stress from rapid socioeconomic changes, sleep disturbances, and environmental factors like air pollution [35,36]. These findings underscore how India's ongoing epidemiological transition is manifesting in worsening metabolic health across all populations, though with distinct urban-rural patterns that demand tailored public health responses addressing both environmental determinants and individual risk behaviors.

Hypercholesterolemia and hypertriglyceridemia: Dyslipidemia, particularly hypercholesterolemia and hypertriglyceridemia, constitutes a significant modifiable risk factor for NCDs, especially CVD, which remains the leading cause of mortality in India. The ICMR-INDIAB study, one of the most extensive investigations into cardiovascular risk factors in the country, revealed a high burden of lipid abnormalities among Indian adults: hypercholesterolemia (≥200 mg/dL) in 24.0% of the population, elevated LDL cholesterol (>130 mg/dL) in 20.9%, low HDL cholesterol in 66.9%, and hypertriglyceridemia in 32.1% [11]. These findings underscore a worrying trend, as dyslipidemia often remains undiagnosed and inadequately treated in both urban and rural populations, contributing silently to the growing burden of ischemic heart disease and stroke. Several regional and national studies echo these patterns. The India Heart Watch study has similarly identified a significant prevalence of lipid abnormalities, with urban populations typically exhibiting higher total cholesterol and triglyceride levels than rural populations due to dietary transitions, physical inactivity, and obesity [37]. Inadequate screening and lack of awareness further exacerbate the challenge, particularly among younger adults who are increasingly exposed to lifestyle risks. Addressing these dyslipidemias through early detection, statin therapy where appropriate, and lifestyle interventions is critical to reversing India’s rising NCD trajectory [32,38].

Converging trend

A comparative analysis of NFHS-4 (2015-16), NNMS (2017-18), and NFHS-5 (2019-21) data reveals a notable convergence in NCD risk factor prevalence between urban and rural populations in India (Figure 1 and Figure 2). Historically, urban areas bore a disproportionate burden of lifestyle-related risk factors; however, recent trends indicate a closing gap. For instance, the prevalence of overweight among rural women increased from 15.0% (NFHS-4) to 19.7% (NFHS-5), while among rural men it rose from 14.3% to 19.3%, indicating a nutritional transition in these populations. Similarly, rural men exhibited a significant rise in hypertension, from 11.8% to 20.5%, while urban prevalence remained relatively stable, suggesting greater vulnerability and fewer control mechanisms in rural health systems. Raised blood sugar prevalence also showed a rising trend among rural women (from 7.5% to 11.4%) and men (from 10.9% to 13.5%), approaching urban levels and reflecting the widespread penetration of diabetogenic risk behaviors into rural areas. Furthermore, behavioral risk factors such as tobacco and alcohol use remained high or increased in rural populations, especially among men, and even showed emerging trends among women, challenging the traditional notion of urban predominance. These patterns point toward a shift in disease epidemiology, with rural India undergoing a rapid epidemiological transition, and highlight the emergence of “double burden zones,” where undernutrition and infectious diseases coexist with rising NCD risks.

Social and structural determinants

The rising burden of NCDs in India is deeply intertwined with social and structural determinants that shape individual behaviors and health outcomes. Literacy plays a pivotal role in enabling health-seeking behavior, understanding risk factors, and adhering to prevention and treatment regimens. Low levels of education, particularly among women in rural areas, are associated with higher exposure to behavioral risks such as tobacco use and unhealthy diets, as well as lower utilization of health services [8,39]. Gender also emerges as a critical determinant. Women in low-resource settings often face intersecting vulnerabilities, including restricted autonomy, caregiving burdens, and poor access to screening and diagnostic services. These inequities contribute to delayed diagnosis and underreporting of conditions such as hypertension and diabetes [40]. In contrast, urban men and youth are increasingly exposed to psychosocial stress, substance use, and sedentary lifestyles, driven by evolving work environments and social norms [6]. Additionally, the awareness, treatment, and control rates for major NCDs such as hypertension and diabetes are consistently lower in rural India, reflecting significant healthcare access gaps [8]. Rural populations often experience inadequate availability of diagnostic services, poor continuity of care, and limited public health outreach. Environmental factors, including pollution, unsafe built environments, and lack of recreational spaces, compound the risk, especially in rapidly urbanizing areas [21].

Discussion

This review highlights the evolving landscape of NCD risk factors in India amidst rapid epidemiological and urban-rural transitions. National datasets such as NFHS-4, NFHS-5, NNMS, and the ICMR-INDIAB study reveal a dual burden of NCD risk factors: behavioral risks such as tobacco and alcohol use continue to be disproportionately higher in rural populations, while metabolic risks, including hypertension, diabetes, and obesity, are more prevalent in urban areas. This risk divergence reflects complex interactions among social determinants, occupational patterns, cultural norms, and systemic access to preventive care. The findings suggest that while urban populations are burdened by the consequences of lifestyle modernization, such as physical inactivity and dietary shifts, rural populations are experiencing a surge in NCD risk due to transitions in behavior and poor access to health infrastructure. Importantly, rural India now faces a convergence of traditional and modern risk factors, with growing rates of overweight and raised blood sugar levels, particularly among women. These patterns signal an impending shift that could erode the historically protective rural health advantage. Furthermore, disparities in awareness, diagnosis, treatment, and control rates across NCDs, especially hypertension and diabetes, underscore the systemic gaps in healthcare access and utilization. Sociodemographic variables such as gender, education, and socioeconomic status further stratify these outcomes. This necessitates an urgent need to move beyond homogenized public health strategies toward geographically nuanced, equity-oriented interventions that reflect the lived realities of both urban and rural populations. To further synthesize these findings, we propose a structured comparative framework (Table 1) that categorizes NCD risk disparities, underlying determinants, and corresponding policy levers across rural and urban India. This framework helps visualize how divergent risk environments require differentiated yet complementary strategies and highlights areas of convergence, such as rising obesity in rural women, where integrated interventions are necessary.

**Table 1:** Comparative framework of NCD risk factors and interventions across rural and urban India A comparative analysis of NCD risk profiles in India reveals distinct yet converging trends between rural and urban populations. Rural areas continue to exhibit high levels of behavioral risk factors, particularly tobacco and alcohol use, driven by socio-cultural and access-related determinants. Conversely, urban populations face a predominance of metabolic risks such as obesity, hypertension, and diabetes, linked to sedentary lifestyles and dietary shifts. Notably, a convergence is emerging, with rising obesity and metabolic conditions now observed in rural, especially peri-urban, populations, indicating a transition toward a dual burden of disease. These evolving patterns underscore the need for differentiated yet integrated policy responses. Rural contexts require intensified health promotion and behavior change strategies, while urban settings demand robust screening programs and structural reforms in urban planning. Cross-cutting approaches, including digital health tools and gender-sensitive interventions, are essential. These findings support the development of an urban-rural transition model to inform risk stratification, contextual intervention design, and policy alignment in India’s NCD response. NCD, non-communicable disease; ASHA, accredited social health activist

Domain	Rural Context	Urban Context	Converging Trends
Dominant Risk Type	Behavioral risks	Metabolic + biochemical risks	Double burden emerging in peri-urban/rural zones
Key Risk Factors	Tobacco use, alcohol use, unhealthy diet	Obesity, hypertension, diabetes, dyslipidemia	Rising obesity in rural women
Determinants	Low literacy, poor access, cultural norms	Sedentary lifestyle, processed food, stress	Urbanization of rural diets
Policy Levers Needed	Health promotion, behavior change, ASHA capacity	Screening, metabolic clinics, urban planning reforms	Digital tools, gender-sensitive strategies

Proposed model: urban-rural NCD risk transition framework in India

This conceptual model illustrates the divergent yet intersecting pathways of NCD risk in India. In rural areas, behavioral risk factors such as tobacco and alcohol use dominate due to entrenched cultural practices, weak regulatory oversight, and low health literacy. Conversely, urban populations exhibit higher levels of metabolic and biochemical risks, driven by sedentary lifestyles, dietary transitions, stress, and environmental exposures. The model captures four domains: dominant risk type, key risk factors, structural and social determinants, and required policy response.

The coexistence of traditional rural risks with emerging urban trends points to a convergence that demands tailored, context-specific interventions. Health systems must adopt differentiated packages of care and leverage community-level engagement in rural areas while promoting lifestyle modification, environmental design, and metabolic screening in urban centers. The framework also emphasizes the role of gender, socio-economic status, and education in shaping NCD risk profiles and highlights the importance of cross-sectoral governance to address these inequities.

Limitations

This review is subject to certain methodological limitations inherent in the use of secondary data. The analysis primarily relies on nationally representative surveys such as NFHS-4 and NFHS-5, NNMS, ICMR-INDIAB, and GATS, which, while robust, vary in their data collection periods, definitions, and scope. These variations may affect the comparability of indicators across time and regions. Moreover, biochemical risk factor data were less comprehensively available than behavioral or metabolic indicators, limiting our ability to analyze certain NCD trends in depth. Additionally, while all figures and tables presented in this review were self-generated from publicly available data, they have not undergone formal peer-review validation.

Policy implications

Context-Specific Public Health Strategies

Rural areas require intensified behavior change communication, tobacco and alcohol cessation services, and community-based health promotion campaigns. Urban regions must prioritize screening for metabolic risks, lifestyle interventions, urban planning that promotes physical activity, and dietary regulation policies.

Differentiated Service Packages Under the National Programme for Prevention and Control of Non-communicable Diseases

Health and Wellness Centers (HWCs) should customize services: addiction counseling in rural PHCs and metabolic management in urban HWCs.

Risk-Based Resource Allocation

Use district-level NCD risk maps to drive targeted investment and personnel deployment. Regions with high behavioral risks need community health worker engagement; those with high metabolic risks require enhanced diagnostics and chronic care capacity.

Gender and Equity Lens

Tailor strategies for rural women, who face rising obesity and limited access to care, and for the urban poor, who are often under-screened and underserved.

Policy and Regulatory Interventions

Expand tobacco and alcohol taxation and ban illicit brews in rural areas. In urban areas, adopt sugar-sweetened beverage taxes, front-of-pack food labeling, and green urban infrastructure to promote healthy lifestyles.

Integrated Surveillance and Evaluation

Strengthen national risk factor surveillance using WHO STEPS tools and harmonize datasets across NFHS, NNMS, and other platforms to inform continuous policy refinement.

By aligning interventions with the risk profiles of India’s diverse populations, policymakers can build a more responsive and resilient public health system capable of combating the NCD epidemic and achieving health equity.

## Conclusions

India’s growing NCD burden reflects not just an epidemiological crisis but a multidimensional public health challenge that is unevenly distributed across urban and rural geographies. The persistence of behavioral risk factors in rural areas, alongside the escalating metabolic risk burden in urban populations, calls for a recalibration of national health strategies. Strengthening primary health care systems, improving surveillance, and tailoring interventions to local contexts are essential to mitigate this dual burden. An integrated, life-course approach grounded in preventive care, behavioral modification, and systems-level policy reform will be pivotal to reversing current trends. Without prompt and context-sensitive action, India risks deepening health inequities and facing unsustainable health and economic costs in the coming decades.
